# A critical threshold of MCM10 is required to maintain genome stability during differentiation of induced pluripotent stem cells into natural killer cells

**DOI:** 10.1098/rsob.230407

**Published:** 2024-01-24

**Authors:** Megan M. Schmit, Ryan M. Baxley, Liangjun Wang, Peter Hinderlie, Marissa Kaufman, Emily Simon, Anjali Raju, Jeffrey S. Miller, Anja-Katrin Bielinsky

**Affiliations:** ^1^ Department of Biochemistry, Molecular Biology, and Biophysics, University of Minnesota, Minneapolis, MN, USA; ^2^ Division of Hematology, Oncology and Transplantation, University of Minnesota, Minneapolis, MN, USA

**Keywords:** DNA replication, induced pluripotent stem cells, MCM10, natural killer cells, telomeres

## Abstract

Natural killer (NK) cell deficiency (NKD) is a rare disease in which NK cell function is reduced, leaving affected individuals susceptible to repeated viral infections and cancer. Recently, a patient with NKD was identified carrying compound heterozygous variants of *MCM10* (*minichromosome maintenance protein 10*), an essential gene required for DNA replication, that caused a significant decrease in the amount of functional MCM10. NKD in this patient presented as loss of functionally mature late-stage NK cells. To understand how MCM10 deficiency affects NK cell development, we generated *MCM10* heterozygous (*MCM10^+/−^*) induced pluripotent stem cell (iPSC) lines. Analyses of these cell lines demonstrated that *MCM10* was haploinsufficient, similar to results in other human cell lines. Reduced levels of MCM10 in mutant iPSCs was associated with impaired clonogenic survival and increased genomic instability, including micronuclei formation and telomere erosion. The severity of these phenotypes correlated with the extent of MCM10 depletion. Significantly, *MCM10^+/−^* iPSCs displayed defects in NK cell differentiation, exhibiting reduced yields of hematopoietic stem cells (HSCs). Although *MCM10^+/−^* HSCs were able to give rise to lymphoid progenitors, these did not generate mature NK cells. The lack of mature NK cells coincided with telomere erosion, suggesting that NKD caused by these *MCM10* variants arose from the accumulation of genomic instability including degradation of chromosome ends.

## Graphical abstract

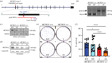

## 1. Introduction

Defects in DNA replication have been linked to multiple congenital diseases that exhibit surprisingly different phenotypes [1]. One such disease is natural killer (NK) cell deficiency (NKD). NK cells are lymphocytes of the innate immune system that have cytotoxic and immunoregulatory roles in tumour surveillance and viral clearance [2]. The predominant model for NK cell development is a linear progression through 5 stages starting with hematopoietic stem cells (HSCs) in the bone marrow, and transitioning through the lymphoid progenitor (LP) stage to be committed to innate lymphoid development at stage 3 in secondary lymphoid tissue [2–4]. The final stages of development similarly take place in secondary lymphoid tissue. However, both stage 4 and 5 NK cells can also be detected in peripheral blood [4–6]. Stage 4 cells have immunomodulatory roles and are identified by high cluster of differentiation (CD) 56 surface expression and absence of T cell marker CD3 [2]. These cells are thought to give rise to the more abundant cytotoxic stage 5 NK cells, which represent approximately 90% of all NK cells in peripheral blood [5–7]. Stage 5 cells are characterized by low expression of CD56 and high expression of CD16 [8]. Generally, NK cells represent between 3 and 30% of the lymphocyte population. However, in classical NKD they are less than 1%, making individuals highly vulnerable to viral infection [9–11]. Importantly, the closely related T and B cell populations are unaffected [12]. Classical NKD has been associated with pathogenic variants of several DNA replication genes, including *minichromosome maintenance protein 4* (*MCM4*), *MCM10*, *go-ichi-ni-san subunit 1* (*GINS1*), and *GINS4*. Individuals with NKD caused by these variants predominantly have significant depletion of terminally differentiated stage 5 NK cells [13–19].

DNA replication is a complex process required for cell division and is conceptually divided into multiple steps: (1) origin licensing, (2) origin firing, (3) elongation and (4) termination. During origin licensing, in G1 phase of the cell cycle, two MCM2-7 complexes are loaded onto the DNA in a head-to-head orientation at each origin [20,21]. During the transition from G1 to S phase, the GINS complex and the helicase co-activator cell division cycle 45 (CDC45) bind to MCM2-7 to form the CDC45:MCM2-7:GINS (CMG) helicase [22,23]. MCM10 is known as a ‘firing factor’ that is essential for CMG activation [22,24–26]. In addition to its role as a firing factor, MCM10 has several additional functions in DNA replication. In yeast, *Xenopus* egg extracts, and human cells, MCM10 is important for recruitment of DNA polymerase α [27–30]. Furthermore, *in vitro* studies have suggested that MCM10 aids the replisome in bypassing bulky lesions [31]. Additional studies have characterized a role for MCM10 in inhibiting fork regression, maintaining fork stability and preventing unrestrained fork progression upon encountering replication stress [30,32–34]. Altogether, these observations suggest that MCM10 plays an important role in both replication initiation and maintenance of efficient DNA synthesis throughout elongation.

Inefficient DNA replication precipitates genomic instability. Telomeres are particularly vulnerable to incomplete replication and are considered ‘difficult-to-replicate’ regions. This is due, in part, to their lack of replication origins and reliance on replisomes initiating in nearby subtelomeric regions [35,36]. If these subtelomeric origins are not activated, cells are reliant on origins of replication even further from the telomere, increasing the probability that they will stall and prevent complete replication of chromosome ends [35]. In addition, as the replisome encounters telomeric DNA, it must replicate through a highly repetitive sequence that is prone to form secondary structures (e.g. G-quadruplexes) [37]. Ultimately, incomplete replication can lead to telomere loss. We recently described increased telomere erosion and other forms of genomic instability in human cell lines that modelled *MCM10* patient variants [38]. Interestingly, these phenotypes were more severe in transformed HCT116 than in non-transformed hTERT RPE-1 cells, suggesting that there are cell type specific MCM10 thresholds for robust maintenance of genome stability [38]. These observations led us to hypothesize that inefficient DNA replication leads to genomic instability during NK cell differentiation that inhibits NK cell development. In this study, we generated *MCM10* heterozygous (*MCM10^+/−^*) induced pluripotent stem cells (iPSCs) to assess telomere maintenance throughout NK cell differentiation.

We show here that *MCM10* is haploinsufficient in iPSCs leading to genomic instability and poor clonogenic survival. We isolated and characterized two clones that expressed different levels of MCM10, allowing us to evaluate the level of genomic instability related to different doses of MCM10. Both *MCM10^+/−^* clones had increased levels of micronuclei, a hallmark of genomic instability, but only the clone expressing less MCM10 exhibited telomere erosion at the iPSC stage. Despite these differences, mutant clones were equally impaired in their ability to generate HSCs and mature NK cells. We assessed telomere length as cells progressed from iPSCs to NK cells and observed a normal degree of telomere erosion in wild-type (WT) cells. However, in MCM10 deficient cells telomere shortening was more significant, leading to an increase in chromosome ends lacking detectable telomere signal, ‘signal-free ends’ (SFE), at the NK cell stage. Taken together, we demonstrate that *MCM10*^+/−^ iPSCs exhibit genomic instability and that the differentiation into NK cells is impacted at two stages, the generation of HSCs and mature stage 5 NK cells.

## 2. Results

### 2.1. *MCM10* is haploinsufficient in induced pluripotent stem cells

**Figure 1.**
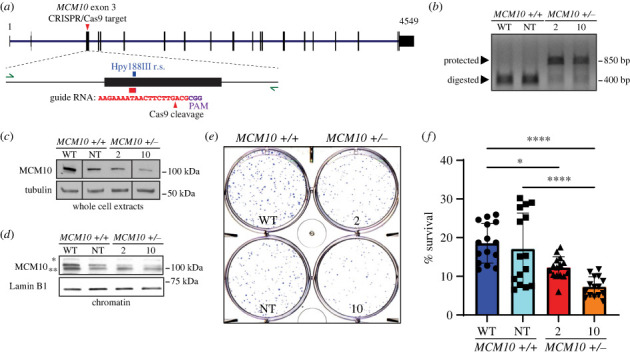
*MCM10* is haploinsufficient in iPSCs. (*a*) Schematic of *MCM10* indicating the location of CRISPR/cas9 gene targeting. Enlarged schematic of exon 3 has green arrows indicating location of primers used for genotyping, a red/purple box indicating the location of the guide RNA, and a blue box indicating the location of the Hpy1888III restriction site that was utilized to assess targeting. Inset contains nucleotide sequence of guide RNA used in gene targeting. (*b*) Genotyping of clones following CRISPR/Cas9 targeting. Heterozygous clones 2 and 10 showed successfully targeted alleles as demonstrated by bands protected from digestion by the Hpy188III. Untargeted alleles in WT and NT cell lines showed complete digestion. (*c*) Representative western blot of MCM10 with tubulin as the loading control from whole cell extracts. These data are from a single experiment where intervening lanes have been removed. (*d*) Representative western blot of MCM10 with Lamin B1 as the loading control from chromatin extracts. Ubiquitinated form of MCM10 is indicated by an asterisk. Degradation product of MCM10 is indicated by two asterisks. (*e*) Representative image of clonogenic survival in which 2000 cells were plated. (*f*) Quantification of clonogenic survival from 5 biological replicates. Each individual point (black symbols) represents a technical replicate (3 per biological replicate). Error is indicated as standard deviation (s.d.). Significance was calculated using one-way ANOVA. *<0.05, ****<0.0001.

We previously reported compound heterozygous variants of *MCM10* that caused NKD and restrictive cardiomyopathy, respectively [18,38]. In these patients, one allele was null and the second was hypomorphic [18,38]. When modelling these variants in transformed HCT116 and non-transformed hTERT-immortalized RPE-1 human cell lines, we demonstrated that *MCM10* was haploinsufficient. Reduction of MCM10 in both cell types resulted in fewer active replication forks, but only HCT116 mutants had a measurable impairment of global DNA synthesis and resulting impact on cell cycle distribution. Despite normal cell cycle distribution, *MCM10^+/−^* RPE-1 cells exhibited a reduction in proliferation and increased apoptosis [18,38]. To understand the effects of MCM10 deficiency during differentiation, we targeted exon 3 of *MCM10* in iPSCs derived from adult female dermal fibroblasts (iPS12-10) with clustered regularly interspaced short palindromic repeats (CRISPR) and CRISPR-associated protein 9 (CRISPR/Cas9) (figure 1*a*). We isolated two *MCM10* heterozygous clones (2 and 10), and one non-targeted clone (NT) that retained two normal *MCM10* alleles (figure 1*b* and electronic supplementary material, figure S1*a*). We included this NT clone to ensure that the observed effects were not due to genome editing *per se*, but to the monoallelic knockout of *MCM10*. Following gene targeting, cell lines retained key iPSC characteristics including octamer-binding transcription factor 4 (OCT4) expression and normal morphology (electronic supplementary material, figure S1*b*).

Similar to our model cell lines, we found that *MCM10^+/−^* iPSCs had a significant reduction in protein expression in comparison to parental WT cells. Moreover, we observed a nearly two-thirds reduction of chromatin bound MCM10 (figure 1*c*,*d* and electronic supplementary material, figure S1*c*,*d*). Interestingly, although mutant clone 10 appeared to have lower MCM10 expression compared to mutant clone 2, both clones maintained a similar level of chromatin bound MCM10. We hypothesized that this reduction of MCM10 would cause defects in cell growth and survival. To assess this, we compared the ability of single cells from the *MCM10^+/−^*, NT and parental WT populations to form colonies. Both *MCM10^+/−^* clones, but not the NT clone, had a significant reduction in clonogenic survival compared to the parental cell line (figure 1*e*,*f*). Together, these results demonstrate that *MCM10* is haploinsufficient in human iPSCs.

### 2.2. Cell cycle distribution appears unaltered in *MCM10* induced pluripotent stem cell mutants

**Figure 2.**
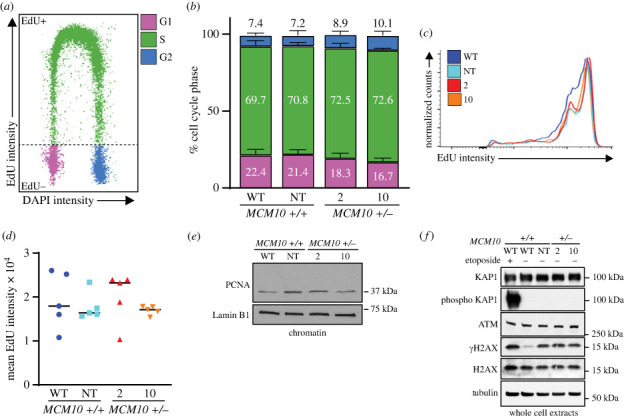
MCM10 depletion does not affect cell cycle distribution. (*a*) Example plot indicating G1, S and G2 phases of the cell as identified by chromatin flow experiments. (*b*) Cell cycle distribution of parental WT, NT and *MCM10^+/−^* cell lines. Error bars indicate s.d. and *n* = 5 biological replicates. There was no significant difference for each cell cycle phase between cell lines as measured by one-way ANOVA. (*c*) Representative comparison of EdU incorporation during S phase for WT, NT and *MCM10^+/−^* cell lines. (*d*) Mean EdU intensity in S phase from 5 biological replicates for WT, NT and *MCM10^+/−^* cell lines. There was no significant difference between cell lines as measured by one-way ANOVA. (*e*) Representative western blot of PCNA with Lamin B1 as the loading control from chromatin extracts. (*f*) Western blot of KAP1, phosphorylated KAP1, ATM, γ-H2AX, H2AX with tubulin as the loading control from whole cell extracts, with or without preceding etoposide treatment.

We previously reported that transformed *MCM10* mutant cells showed a reduction in the rate of DNA synthesis and a decrease in the S phase population. However, that was not true for non-transformed mutant cells, implying that the latter were less sensitive to MCM10 depletion [38]. Moreover, during the generation of iPSCs dynamics of DNA replication can change dramatically, including variations in fork speed and the number of active replication origins [39–41]. Thus, we were curious as to whether iPSCs behaved similarly to highly proliferative transformed or non-transformed cells [32]. We used quantitative chromatin flow cytometry to assess cell cycle distribution, origin licensing and the rate of DNA synthesis. To perform these analyses, we pulse-labelled iPSCs with 5-ethynyl-2′-deoxyuridine (EdU) for 30 min followed by isolation of nuclei and extraction of proteins not bound to chromatin. We then stained for EdU, MCM2 and total DNA content with 4′,6-diamidino-2-phenylindole (DAPI). The combination of DAPI staining and EdU labelling allowed for precise identification of replicating cells and staining of chromatin bound MCM2 was used to assess origin licensing (figure 2*a*). We confirmed that the *MCM10^+/−^* iPSC clones maintained normal licensing, indicated by equivalent levels of MCM2 loading in G1 phase when compared to WT (electronic supplementary material, figure S2*a*,*b*). The cell cycle distribution was not significantly different between the *MCM10^+/−^* iPSC clones and either parental WT or NT cells, nor did we see any differences in DNA synthesis when comparing maximal or mean EdU intensities (figure 2*a–c*). Consistent with these results, all cell lines had similar amounts of chromatin bound proliferating cell nuclear antigen (PCNA), a critical DNA polymerase processivity factor present at all replication forks (figure 2*d*). These results were similar to those previously reported for *MCM10^+/−^* non-transformed RPE-1 cells [18,38]. It is worthwhile to note that both mutant iPSCs and RPE-1 cells nevertheless displayed significant proliferation defects. Finally, to understand if *MCM10^+/−^* iPSC clones experience increased DNA double stranded breaks (DSBs), we performed western blot analyses for phosphorylated KRAB-associated protein-1 (KAP1) and phosphorylated histone H2AX (*γ*H2AX; figure 2*f*). KAP1 is a target of the Ataxia-telangiectasia mutated (ATM) kinase and histone H2AX is a target of both the Ataxia telangiectasia and Rad3 related (ATR) and ATM kinases. While phospho-KAP1 showed a strong signal in etoposide treated WT iPSCs, it was undetectable in all untreated cell lines independent of *MCM10* status. Interestingly, untreated NT or *MCM10^+/−^* iPSC clones showed elevated *γ*H2AX levels in comparison to WT, although this remained lower than in etoposide treated WT iPSCs. Taken together, these data imply that MCM10 deficient iPSCs did not generate DSBs. It is important to note that the level of global replication stress as indicated by *γ*H2AX levels was identical in the NT and *MCM10^+/−^* iPSC clones. This implies that phenotypic differences between MCM10 proficient or deficient iPSCs cannot simply be attributed to an increase in general replication stress.

### 2.3. *MCM10*^+/−^ induced pluripotent stem cells exhibit decreased survival due to increased genomic instability

**Figure 3.**
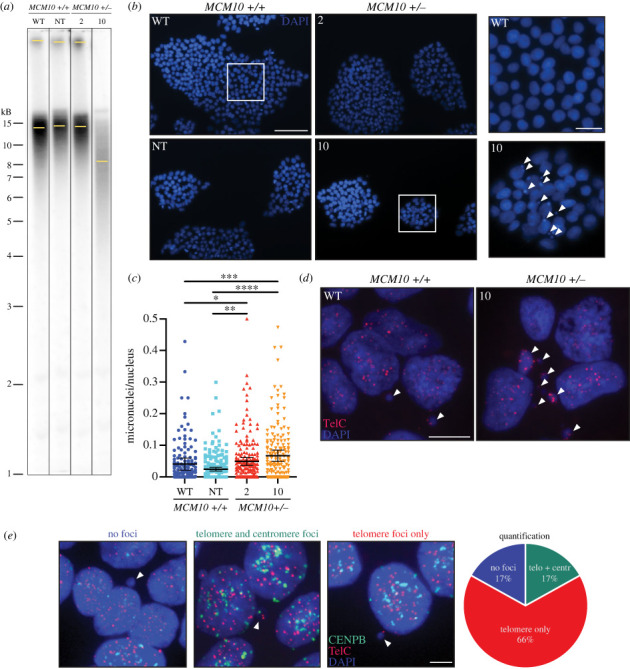
Genomic instability arises in a dose dependent manner after MCM10 depletion. (*a*) Representative TRF analysis of WT and *MCM10^+/−^* cell lines. Yellow bars indicate intensity peaks in each lane. (*b*) (Left) Example images with DAPI staining utilized to visualize nuclei and micronuclei. Scale bar is 100 µm. (Right) Magnified fields of DAPI stained nuclei. White arrows indicate micronuclei. Scale bar is 50 µm. (*c*) Quantification of micronuclei. Data are representative of 7 biological replicates. For display purposes the maximum value of the *y*-axis is set to 0.5 causing six data points to be outside the axis limit. The full dataset is displayed in electronic supplementary material, figure S3D. Error bars indicate 95% confidence intervals. Data were determined not to be normally distributed by D'Agostino and Pearson test. Significance was determined by Kruskal–Wallis test. *<0.05, **<0.01, ***<0.0005, ****<0.0001. (*d*) Example images with DAPI (blue) staining and T-FISH (red, TelC) of interphase nuclei. White arrows indicate micronuclei containing telomeric DNA. Scale bar is 5 µm. Example images with DAPI (blue) staining with centromere- (green, CENPB) and T-FISH (red, TelC) of interphase nuclei. White arrows indicate micronuclei from *MCM10^+/−^* clone 10 with either no foci, telomere foci only, or both telomere and centromere foci. The percent of each class is shown in the pie chart (*n* = 124 total micronuclei). Scale bar is 5 µm.

Chronic replication stress has cumulative effects over time and is not always evident in a single cell cycle ‘snapshot’. Since we did not observe cell cycle abnormalities, we hypothesized that our *MCM10^+/−^* clones had reduced survival due to chronic replication stress undetectable by flow cytometry. Both telomere erosion and micronuclei formation are hallmarks of genomic instability that may arise from replication stress [42]. In addition, we previously demonstrated that MCM10 deficient cell lines are prone to telomere erosion [38]. When we used telomere restriction fragment (TRF) assays to interrogate telomere length in the iPSC cell lines, we observed a reduction in average telomere length from approximately 13 kb in parental WT cells to 7.5 kb in mutant clone 10 but did not see a reduction in NT or mutant clone 2 cells (figure 3*a* and electronic supplementary material, figure S3*a*). As a complementary approach, we used telomere fluorescence *in situ* hybridization (T-FISH) followed by quantitative measurement of fluorescence intensities on metaphase spreads to examine changes in telomere length. One key advantage of this method is that it requires fewer cells. To confirm the accuracy of this assay we utilized three HCT116 cell lines including WT, WT overexpressing ‘super-telomerase’ (ST), and *MCM10^+/−^* clone 8 [43]. We previously used TRF analyses to demonstrate that HCT116 ST telomere length is greater than 12 kb, that of HCT116 WT is 5–6 kb and of HCT116 *MCM10^+/−^* clone 8 is 2–4 kb [38]. Consistently, when we assessed telomere signals on metaphase spreads, ST cells had average signal intensities 1.9-fold higher than WT. WT intensities, in turn, were on average 1.7-fold higher than those of HCT116 *MCM10^+/−^* clone 8, in agreement with our previous studies (electronic supplementary material, figure S3*b*). These results demonstrate that quantitative measurement of telomere fluorescence on metaphase chromosomes recapitulates telomere measurements using TRF analysis. We therefore used quantitative T-FISH to measure relative telomere length in iPSCs. Interestingly, mutant clone 10 had a significant reduction of average telomeric signal compared to WT, NT and mutant clone 2. The small but statistically significant reduction in average telomere signal intensity for mutant clone 2 and the NT clone in comparison to parental WT cells was likely due to telomere erosion associated with normal cell culturing, as these two clones were not different from each other (electronic supplementary material, figure S3*c*). These results were consistent with the TRF analysis of the same cell lines (electronic supplementary material, figure S3*c*).

Telomere erosion is not the only measure of genomic instability. We also quantified micronuclei, which occur as a result of defects in chromosome segregation, nuclear envelope assembly or through generation of acentric chromosomes [44]. To this end, we counted the number of micronuclei per iPSC colony and normalized it to the number of nuclei in each colony. We observed a significant increase in micronuclei in both *MCM10^+/−^* clones (figure 3*b*,*c* and electronic supplementary material, figure S3*d*). Interestingly, a subset of micronuclei for both WT and *MCM10^+/−^* cells contained telomeres, as indicated by T-FISH (figure 3*d*), suggesting that telomere erosion and micronuclei formation might be connected. To further evaluate whether micronuclei formation is linked to telomere defects in *MCM10* mutant iPSCs, we performed FISH analyses for both telomeres and centromeres in *MCM10^+/−^* clone 10 (figure 3*e*). The majority of micronuclei (83%) contained telomere foci, with a subset of those also containing centromere foci (20% of telomere positive micronuclei, and 17% of all micronuclei). Notably, we did not observe micronuclei containing only centromere foci. In addition, a fraction of micronuclei contained diffuse or ‘hazy’ centromere FISH signals, suggesting that centromeric DNA in these micronuclei was under-condensed (electronic supplementary material, figure S3*e*). Although elevated telomere erosion and micronuclei formation did not lead to a significant increase in apoptosis, measured by Annexin V expression (electronic supplementary material, figure S3*f*), they are clear indicators of increased genomic instability which—over multiple cell divisions—likely culminated in poor clonogenic survival of *MCM10^+/−^* iPSCs.

### 2.4. MCM10 deficiency impacts multiple steps of natural killer cell differentiation

**Figure 4.**
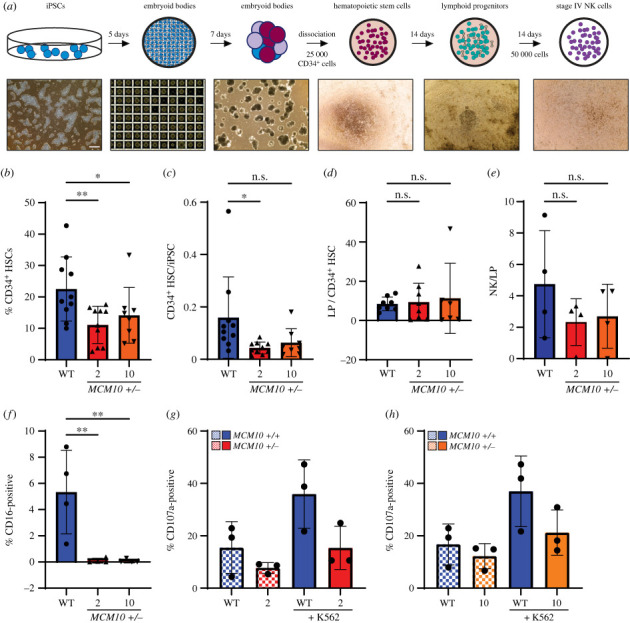
MCM10 deficiency disrupts NK cell differentiation. (*a*) Differentiation schematic that begins with iPSCs (blue) which are dissociated into single cells and plated into microwells. Over five days EBs form. These EBs are transferred to a non-treated 6-well tissue culture plate to further develop for another seven days. After a total of twelve days the EBs are dissociated, and CD34^+^ HSCs are isolated. HSCs are plated at a density of 25 000 cells/well and differentiated into LPs for fourteen days. Cells are then harvested and plated at a density of 50 000 cells per well and differentiated into NK cells. For more details on this process, see Material and methods. (*b*) Percentage CD34^+^ cells in the live population following dissociation of EBs. Error is indicated as s.d. Each black symbol indicates one biological replicate. Significance was determined by one-way ANOVA. *<0.05, **<0.01. (*c*) Number of CD34^+^ cells generated per iPSC plated. Error is indicated as s.d. Each black symbol indicates one biological replicate. Significance was determined by one-way ANOVA. *<0.05, n.s. = not significant. (*d*) Number of cells generated at collection during LP differentiation per CD34^+^ cell plated. Error is indicated as s.d. Each black symbol indicates one biological replicate. There was no significant difference as determined by one-way ANOVA. (*e*) Number of NK cells (CD3^−^, CD56^+^) generated per LP cell plated. Error is indicated as s.d. Each black symbol indicates one biological replicate. There was no significant difference as determined by one-way ANOVA. (*f*) Percentage of CD16^+^ cells within the NK cell population. Error is indicated as s.d. Each black symbol indicates one biological replicate. Significance was determined by one-way ANOVA. **<0.01. (*g*,*h*) Percentage of CD107^+^ cells within each NK cell population from clones 2 and 10. Each black symbol indicates one biological replicate. Error is indicated as s.d. There was no significant difference as determined by one-way ANOVA.

Because NKD is caused by compound heterozygous variants in *MCM10*, we wanted to explore the ability of *MCM10^+/−^* mutant iPSCs to differentiate into NK cells. To achieve this, we utilized a protocol in which iPSCs were seeded in a microwell plate. Within each well an embryoid body forms containing CD34^+^ HSCs with supporting stromal cells. After 12 days, these embryoid bodies were dissociated and the HSC population was isolated. HSCs were then plated and further differentiated for 14 days into LPs. These were collected and plated for differentiation over an additional 14 days into NK cells (figure 4*a*). The predominant cell type derived from this *in vitro* differentiation protocol are stage 4 NK cells [45–47]. However, we were able to generate a small population of stage 5 NK cells, allowing us to assess the effect of MCM10 deficiency on all stages of NK cell development.

Prior to purification of CD34^+^ HSCs and differentiating HSCs into LPs, we sampled WT and mutant populations to compare the production of CD34^+^ HSCs from parental WT or *MCM10^+/−^* iPSCs. We observed a significant reduction of the CD34^+^ HSC population in both *MCM10* mutant cell lines (figure 4*b*). This resulted in a strong reduction in the number of CD34^+^ HSCs produced per iPSC in clones 2 (73% less) and 10 (60% less) (figure 4*c*). Notably, total cells produced per seeded iPSC were equivalent following embryoid body dissociation in *MCM10^+/+^* or *MCM10^+/−^* populations (electronic supplementary material, figure S4*a*). This suggests that the reduction in CD34^+^ cells was not due to reduced cell proliferation, but defective differentiation into CD34^+^ HSCs. When HSCs were directed towards LP differentiation, we did not observe a change in the total number of cells produced per HSC (figure 4*d*). Similarly, there was not a significant reduction in the number of CD56^+^ CD3^+^ NK cells which could be either stage 4 or 5 at the completion of the differentiation protocol (figure 4*e*). Conversely, there was essentially no production of mature stage 5 NK cells from either *MCM10^+/−^* iPSC clone (figure 4*f*). We assessed cytokine production and surface CD107a expression, a measurement for NK cell activation that correlates with cytokine secretion and killing potential of stage 4 and stage 5 NK cells in the presence of K562 target cells [48]. We did not see a significant difference in surface CD107a or TNF*α* and IFN*γ* production between WT and *MCM10^+/−^* derived stage 4 NK cells. This suggests that these *MCM10^+/−^* stage 4 NK cells were functional (figure 4*g*,*h* and electronic supplementary material, figure S4*b–e*). In summary, our *in vitro* differentiation experiments revealed that although *MCM10^+/−^* iPSC clone 10 exhibited a more severe genomic instability phenotype than clone 2, the ability of *MCM10* mutants to differentiate into mature stage 5 NK cells was severely impacted in both clones. Based on our data, loss of MCM10 affected differentiation as early as the formation of HSCs and culminated in the lack of mature stage 5 NK cells.

### 2.5. Defects in natural killer cell development are accompanied by telomere erosion

**Figure 5.**
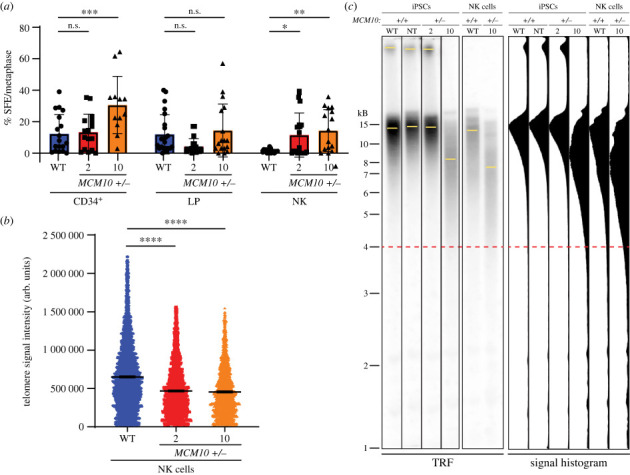
Genomic instability increases during NK cell development. (*a*) Percentage of SFEs per metaphase in CD34^+^ (left), LP (centre) and NK (right) cell populations. Error is indicated as s.d. Each black symbol indicates one metaphase spread. Outliers were determined using the ROUT method. For CD34^+^ and NK cell populations, significance was determined by one-way ANOVA, with *<0.05, **<0.01, ***<0.001, and n.s. = not significant. For LP populations, data were determined not to be normally distributed by D'Agostino and Pearson test. Significance was determined by Kruskal–Wallis test and n.s. = not significant. (*b*) Relative fluorescence of individual telomeres on NK cell metaphase spreads. Data represent telomeres measured from three biological replicates including at least five metaphase spreads each. Error bars indicate 95% confidence interval. Data were determined not to be normally distributed by D'Agostino and Pearson test. Outliers were determined using the ROUT method. Significance was determined by Kruskal–Wallis test, with ****<0.0001. (*c*) TRF analysis in WT, NT and *MCM10^+/−^* iPSCs (same as in figure 3*a*), compared with TRF analysis in WT and *MCM10^+/−^* NK cells. These data are from a single experiment. Yellow bars indicate intensity peaks in each lane. Red dotted line at 4 kb is included to highlight TRF signals in the range of critically short telomeres. Signal intensity scans of each lane are displayed on the right.

We hypothesized that the loss of mature stage 5 NK cells is accompanied by accumulation of genomic instability. Because our previous work showed that loss of MCM10 can lead to accelerated telomere erosion, we set out to examine telomere length in the different stages of NK cell development *in vitro*. We utilized T-FISH on metaphase spreads to measure average telomere signal intensity and quantify SFEs. SFEs are chromosome ends with undetectable telomere signal that is indicative of a critically short telomere. This is a particularly informative measure because a single critically short telomere, rather than shorter average telomere length as measured by TRF, can trigger cell cycle arrest and apoptosis [49]. We first looked at the development of iPSCs to CD34^+^ HSCs. We observed that HSCs derived from *MCM10^+/−^* iPSC clone 10 had more than double the number of SFEs and significantly reduced average telomere signal compared to parental WT cells (figure 5*a* and electronic supplementary material, figure S5*a*). However, HSCs derived from clone 2 did not have a difference in SFEs compared to parental WT controls (figure 5*a* and electronic supplementary material, figure S5*a*). This was surprising because both clones had defects in HSC differentiation. The reduced generation of HSCs may therefore be driven by genomic instability at other regions of the genome. When we evaluated cells differentiated into LPs, clone 10 had reduced average telomere intensity compared to WT (electronic supplementary material, figure S5*b*). However, neither *MCM10^+/−^* clone had a significant increase in SFEs compared to WT LPs (figure 5*a*). Altogether this suggests that MCM10 deficiency has a minor impact on telomere stability during differentiation from iPSC to HSCs and subsequently to LP cells.

Finally, we utilized both quantitative T-FISH and TRF analyses to assess telomere length in NK cells. WT NK cell populations were a mixture of stage 4 and 5 cells, whereas *MCM10^+/−^* NK cells were exclusively stage 4. Metaphase analysis of *MCM10^+/−^* NK cells derived from clones 2 and 10 demonstrated a reduction in average telomere signal by approximately 30% for each population (figure 5*b*). Importantly, TRF data for iPSC clone 10 and its derived NK cells showed that telomeres not only shortened during *in vitro* differentiation but exhibited greater length heterogeneity as indicated by the broad signal extending from 15 kb to below 3 kb (figure 5*c*). This implies that some of these cells have very short telomeres. Unfortunately, we were unable to collect enough *MCM10^+/−^* iPSC clone 2 derived NK cells to perform a TRF analysis. However, we measured SFEs for NK cells generated from clones 2 and 10, and each displayed an approximately 10-fold increase in telomere loss compared to WT NK cells (figure 5*a*). Together, these data show that early steps in NK cell differentiation up to the stage of HSCs were impacted by genomic instability that cannot be explained by telomere shortening alone. However, subsequent differentiation steps induced accelerated telomere erosion in *MCM10^+/−^* mutants. This telomeric loss appeared to predominately take place past the differentiation into LPs and during NK cell maturation. These observations suggest that later stage NK cell development is sensitized to defects in telomere replication. This is consistent with the patient phenotype in which only the NK cell lineage is affected by MCM10 deficiency while other closely related lymphoid lineages remain intact.

## 3. Discussion

MCM10 is critical for efficient DNA replication and its acute depletion results in severe genomic instability [50–52]. We have previously demonstrated that chronic MCM10 deficiency leads to increased cell death, the accumulation of genomic aberrations and telomere maintenance defects [38]. This study extends these results by recapitulating the reduced viability, elevated genomic instability and haploinsufficiency in human iPSCs [38]. A single null allele of *MCM10* is not sufficient to generate disease in humans, nor does it impact mouse development [18,38,53]. Rather, both alleles of *MCM10* must either produce a severely reduced level of WT MCM10 or a hypomorphic form of MCM10 [18,38]. In this study, we selected two mutant iPSC clones that expressed different levels of MCM10*.* Both mutations significantly reduced cell survival, and the severity of telomere erosion correlated with the level of MCM10 depletion. These observations explain why biallelic patient variants caused NKD, but heterozygous parents remained unaffected [18].

Both *MCM10^+/−^* iPSC clones had similar amounts of chromatin bound MCM10 despite differences in total MCM10 expression. Yet, the clone that showed a higher degree of depletion also displayed more severe hallmarks of genome instability. One explanation for the differences seen between these clones is that a depleted pool of unbound MCM10 limits the exchange kinetics when MCM10 dissociates from chromatin. This may not affect origin firing but could impact replisome progression through ‘hard-to-replicate’ regions of the genome, including telomeres. Several studies have suggested that low levels of MCM10 are required for origin firing, while MCM10's role during elongation is dependent on higher protein pools [27,30,54,55]. Furthermore, there is evidence in yeast that multiple copies of Mcm10 are present at each replication fork [26,27]. The role of MCM10 during elongation includes recruitment of DNA polymerase *α*, bypassing lagging strand blocks and stabilizing the replication fork [27–30,34]. Yeast, *Xenopus,* and human MCM10 are each capable of dimerizing *in vitro* and *in vivo* [56–59], aiding in serving as a scaffold for its multiple protein binding partners [58,60]. Through protein interactions, MCM10 has been implicated in preventing replication fork regression as well as unrestrained re-priming upon encountering replication stress [30,32,33]. In yeast, the N-terminal coiled coil domain of Mcm10, which mediates self-association, is needed to elicit a robust response to certain types of replication stress, but it remains unclear whether this function is conserved in human cells [61]. A reduction in free MCM10 could affect both transient association with the replication fork and the protein's propensity to self-interact.

Replication elongation through telomeres poses inherent challenges to replisomes due to their propensity to form secondary structures that can inhibit replication fork progression [36,62]. We previously demonstrated in HCT116 and RPE-1 cells that a reduction of MCM10 expression below 50% resulted in telomere erosion. In our iPSC lines this was not the case; instead more severe depletion of MCM10 was required to elicit telomere erosion. These data support the notion that cell type specific thresholds for MCM10 expression exist, causing different levels of replication stress and telomere instability. Telomere erosion in *MCM10^+/−^* HCT116 cells was due to premature fork stalling within telomeric DNA [38]. We suspect a similar phenomenon is occurring in *MCM10^+/−^* iPSCs at a rate that is insufficient for checkpoint activation. This allows cells with under-replicated DNA to escape into mitosis where stalled replication forks can lead to chromosome breaks and mis-segregation, known causes of micronuclei formation. If forks stalled in telomeres, we would expect to see chromosome fragments with telomeric sequences packaged into micronuclei [44]. Indeed, we observed telomere fragments within the vast majority (83%) of micronuclei in *MCM10^+/−^* clone 10 suggesting that the formation of most micronuclei was linked to telomere maintenance defects when MCM10 levels were significantly reduced.

We believe the telomere erosion phenotype is critical to the tissue specific pathologies seen in *MCM10^+/−^* patients, although replication of other genomic loci, for instance common fragile sites, is presumably also affected by MCM10 deficiency [38,63]. This hypothesis is supported by evidence in the literature. First, pathogenic variants in *regulator of telomere length 1* (*RTEL1*), a binding partner of MCM10 [64,65], have also been reported in NKD patients. RTEL1 is well characterized as a telomere maintenance protein and pathogenic variants of *RTEL1* are also linked to telomeropathies [66–68]. *RTEL1*-linked telomeropathies exhibit earlier onset of NK and combined NK and B cell deficiencies [68]. In addition, while telomeres were not directly assessed, fibroblasts isolated from NKD patients with *GINS1* and *MCM4* pathogenic variants demonstrate increased genomic instability [16]. *Interferon regulatory factor 8* (*IRF8*) pathogenic variants can also lead to NKD, and although this transcription factor regulates expression of many proteins, its notable targets include telomerase and other replication proteins [69–71]. Finally, other hematopoietic lineages may overcome excessive telomere erosion through the upregulation of telomerase or through telomerase independent mechanisms, as was recently described in T cells, thus making them less sensitive to telomere erosion compared to NK cells [45,72–78].

We have demonstrated that *MCM10^+/−^* iPSCs are haploinsufficient and display significant genomic instability. Differentiation places cells under considerable stress, often requiring multiple rapid cell divisions. We previously reported the inability of MCM10 deficient cells to differentiate into NK cells *in vitro* and *in vivo,* but did not assess telomere length or other genomic instability markers during the course of this differentiation process [18]. Additionally, we did not interrogate NK cell differentiation from iPSCs at each transition point (HSC and LP). Here, we demonstrate that *MCM10^+/−^* iPSCs show impaired generation of HSCs. This defect in HSC development was surprising because we did not observe significant perturbations of other blood cell lineages in NKD patients [12,18]. Interestingly, Cacialli *et al.* recently reported that Mcm10 is critical for HSC emergence in zebrafish embryos, suggesting that the protein has an evolutionarily conserved role in hematopoiesis [79]. We also reported a second more severe combination of human variants in *MCM10* that caused fetal demise due to cardiomyopathy and underdeveloped thymus and spleen, consistent with the idea that cell type specific requirements for the level of MCM10 expression exist. Interestingly, while both *MCM10^+/−^* iPSC clones 2 and 10 displayed defects in HSC generation, only clone 10 had measurable differences in telomere length and signal free ends. This indicates that telomere erosion is not driving the defect in HSC generation, although we cannot exclude that these cells may experience replication stress at telomeres without detectable erosion. When we directed an equal number of HSCs to LP differentiation, we harvested similar cell numbers in WT and *MCM10^+/−^* cultures. However, these cells were not analysed for surface markers and the mutants may have a different proportion of LPs than WT controls. Most notably, we were unable to yield any stage 5 NK cells from MCM10 deficient LP populations, arguing that their differentiation potential was severely compromised. These data not only recapitulate the NKD patient phenotype but indicate that changes in each cell type's ability to tolerate telomere instability impacts the differentiation of *MCM10* mutants along the NK cell lineage.

**Figure 6.**
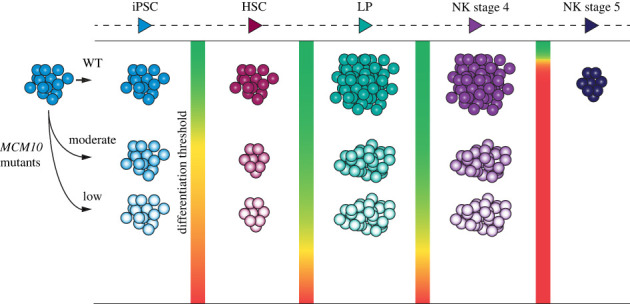
MCM10 deficiency impacts differentiation throughout the NK cell lineage. MCM10 expression affects the ability of iPSCs to differentiate through multiple stages (left to right: HSC, LP, NK stage 4, NK stage 5) and generate mature NK cells. Normal expression in WT cells is indicated in the top row, and lower expressing cells (e.g. *MCM10* mutants) are represented in the middle and bottom rows. At the transition between each developmental stage, we propose a differentiation threshold (indicated by the green to red colour gradient between each column) that determines the number of cells that can develop from a single precursor. The threshold at the iPSC to HSC transition reduces the number of *MCM10* mutant HSCs that can be produced. MCM10 deficiency does not impair differentiation from HSC to LP or LP to NK stage 4. However, the extremely high threshold from stage 4 to 5 NK cells is insurmountable for *MCM10* mutants. This final stage of development corresponds to significant telomere erosion in *MCM10* mutants, which we propose is the cause for the complete block in differentiation.

Few studies have assessed telomere length in NK cells. During normal development, NK cells, like any other cell type, experience a steady decrease of telomerase expression and continuous telomere shortening [75]. However, previous studies relied on assessing telomere length in NK cells that were acquired from peripheral blood or were already at stage 4 and 5. In our study, we analysed telomere maintenance at distinct stages of differentiation from iPSCs to mature NK cells. We demonstrated that NK cells derived from WT iPSCs have shorter telomeres than their parental stem cells, consistent with the idea of steady erosion over time. Moreover, we observed telomere shortening in *MCM10^+/−^* iPSCs differentiating into NK cells. Despite differences in telomere length at the HSC stage, both *MCM10^+/−^* clones had a significant increase in SFE and reduction in telomere signal as cells transitioned from LPs into NK cells, demonstrating this stage of development is particularly sensitive to MCM10 depletion secondary to telomere loss. Importantly, within the *MCM10^+/−^* population we saw greater heterogeneity in telomere length by TRF analysis, and increased SFEs. This is meaningful because one critically short telomere can activate a DNA damage response and lead to cell death or senescence [49]. The dramatic increase in SFEs in stage 4 *MCM10^+/−^* NK cells suggests that the lack of CD16-positive stage 5 NK cells is due to critically short telomeres. Thus, our *in vitro* system recapitulates the patient phenotype and provides a mechanism for the absence of mature stage 5 NK cells. In our model (figure 6) we propose that at each stage of differentiation a minimal amount of MCM10 is required. However, this threshold of MCM10 is variable during different stages of NK cell development, and manifests as an oscillating differentiation threshold. When MCM10 levels are insufficient, telomere-driven genomic instability impairs differentiation. This is compounded by progressive telomere erosion with each proliferative cycle, such that the differentiation threshold becomes insurmountable in *MCM10* mutants at the transition from stage 4 to stage 5 NK cells.

## 4. Material and methods

### 4.1. Cell culture

The iPS12-10 cell line was derived from adult female dermal fibroblasts (Cell Applications, RRID:CVCL_C7XH) and was cultured on tissue culture treated plates (6-well Costar 3506, 24-well Costar 3524) coated with Geltrex (ThermoFisher A1413202) diluted 1:100 in Ham's F12 (Corning 10-080-CV) or Vitronectin XF (StemCell Technologies 100-0763) diluted 1:25 in CellAdhere Dilution buffer (StemCell Technologies 07183), in mTeSR PLUS (StemCell Technologies 05825) with daily medium changes. For passaging, iPSCs were dissociated briefly with Accutase (Innovative Cell Technologies AT104-500) to maintain 3 to 5 cell aggregates. When cells were thawed, they were cultured with 10 µm Y-27632 dihyrochloride (ROCKi) (R&D 1254) for 1 day. HCT116 (ATCC, CCL-247, RRID:CVCL_0291) cells were cultured in McCoys 5A medium supplemented with 10% FBS (Sigma F4135), 1% Pen Strep (Gibco 15140), and 1% L-glutamine (Gibco 205030). Cells were cultured at 37°C and 5% CO_2_.

### 4.2. Cell line generation using CRISPR/Cas9

To genetically engineer iPSCs, cells were dissociated into single cells and 250 000 cells were transfected with 1 µg Cas9 mRNA and 1 µg guide RNA targeting exon 3 of *MCM10* (Synthego Corporation, 5′GAAGAAAATAACTTCTTGACG) in Resuspension Buffer T (ThermoFisher) by electroporation with the Neon Transfection system (1100 V, 20 ms, 1 pulse, 10 µl tip, ThermoFisher MPK1025). Cells were grown with 10 µm ROCKi for 2 days before resuming normal culturing conditions. Single clones were isolated by plating transfected cells at low density and allowing colonies to form. Colonies were then transferred to separate wells by scraping. To assess targeting, primers to introns 2 and 3 of *MCM10* (forward: 5′ GGAGACAAGGAGAACAAAGACC; reverse: 5′ GCTGGCCCAAACATTTCATC) were used to amplify across exon 3 and the PCR product was digested with HPY188III (NEB R0622). Protection from digestion indicated one allele had an insertion/deletion or point mutation disrupting this restriction site and Sanger sequencing was utilized to confirm a mutation that would result in a null allele.

### 4.3. Protein extraction and western blotting

Cells were dissociated, counted and 300 000 cells were plated per well of a 6-well plate in medium containing ROCKi. After 24 h the medium culturing returned to usual. Once approximately 60% confluent, cells were washed in PBS, pelleted, and stored at −80°C. WT and mutant cells were collected at similar confluency and colony size. For etoposide treatment, WT cells were cultured with 10 µM etoposide (RPI E55500) for 2 h. For whole cell extracts, cells were lysed in NETN buffer (0.005% NP-40, 1 mM EDTA, 20 mM Tris-HCl, pH 8.0, 100 mM NaCl) with protease inhibitors (leupeptin, phenylmethylsulfonyl fluoride, *N*-ethylmaleimide, and pepstatin) or in RIPA buffer (50 mM Tris-HCl, pH 8.0, 150 mM NaCl, 10 mM NaF, 1% NP-40, 0.1% SDS, 0.4 mM EDTA, 0.5% sodium deoxycholate, 10% glycerol) for 10 min while rotating. Soluble lysates were isolated by centrifugation at 16 000*g* for 10 min. Proteins were denatured by boiling in the presence of SDS for 5 min and then separated by molecular weight on SDS-PAGE gel and analysed by western blot. For chromatin fractionation, extracts were prepared by lysis in Buffer A (10 mM HEPES, pH 7.9, 10 mM KCl, 1.5 mM MgCl_2_, 0.34 M sucrose, 10% glycerol, 0.1% Triton X-100 and protease inhibitors). Insoluble nuclear proteins were isolated by centrifugation and chromatin-bound proteins were subsequently released by sonication after being resuspended in TSE buffer (20 mM Tris-HCl, pH 8.0, 500 mM NaCl, 2 mM EDTA, 0.1% SDS, 0.1% Triton X-100 and protease inhibitors). The remaining insoluble factors were cleared by centrifugation before fractionation by SDS-PAGE and analysed by western blot. Primary antibodies were diluted in 5% BLOT-QuickBlocker (G-Biosciences 786-011) as follows: rabbit anti-Mcm10 (Novus H00055388-D01P, RRID:AB_11047378; 1:500) mouse anti-GAPDH (GeneTex GTX627408, RRID:AB_11174761; 1:5000), mouse anti-Tubulin (Sigma T9026, clone DM1A, RRID:AB_477593; 1:10 000), mouse anti-PCNA (Abcam ab29, RRID:AB_303394; 1:3000), rabbit anti-Lamin B1 (Proteintech 12987-1-AP, RRID:AB_2136290; 1:3000), rabbit anti-ATM (Proteintech 27156-1-AP, RRID:AB_2880780; 1:1000), mouse anti-KAP1 (Proteintech 66630-1-IG, RRID:AB_2732886; 1:5000), rabbit anti-phosphorylated KAP1 (Abcam ab133440; 1:1000), rabbit anti-H2AX (Bethyl A300-083A, RRID:AB_203289; 1:10 000), rabbit anti-γ-H2AX (Bethyl A300-081A, RRID:AB_203288; 1:5000). Secondary antibodies goat anti-mouse HRP conjugate (Jackson Laboratories 115-035-003, RRID:AB_10015289) and goat anti-rabbit HRP conjugate (Jackson Laboratories 111-035-144, RRID:AB_2307391) were diluted in 5% BLOT-QuickBlocker at 1:10 000. Detection was performed using a WesternBright Quantum detection kit (K-12042-D20) and quantification was done with FIJI version 1.8.0_172 [80].

### 4.4. Immunofluorescence

Two days prior to staining, cells were dissociated, counted and 50 000 cells were plated per well of a 24 well plate in medium containing ROCKi. After 24 h, medium was exchanged for fresh medium without ROCKi. After 24 h, cells were fixed with 4% paraformaldehyde (Electron Microscopy Sciences 15714) for 15 min at room temperature and permeabilized with 0.1% Triton X-100 for 15 min at room temperature. Cells were blocked in ABDIL (20 mM Tris, pH 7.5, 2% BSA, 0.2% fish gelatin, 150 mM NaCl, 0.1% sodium azide) for 1 h at room temperature. Anti-OCT4 (Abcam ab200834, RRID:AB_2924374) was diluted 1:500 in ABDIL and was applied to the slides overnight at 4°C. Cells were washed 3 times with PBST (0.1% Tween in PBS) before applying the secondary antibody (AlexaFlour 488 donkey anti-rabbit, Invitrogen A21206, RRID:AB_2535792) diluted in ABDIL at 1:1000 for 1 h at room temperature. Cells were washed 3 times with PBST, where the second wash contained 5 µg ml^−1^ DAPI (Life Technologies D1306, RRID:AB_2629482). Samples were imaged with an EVOS FL imaging system (ThermoFisher AMF43000). For OCT4 imaging, colonies were first located in the DAPI channel. For micronuclei images, colonies were first located in the phase channel to prevent any bias in choosing colonies. For micronuclei quantification, images were blinded, and micronuclei and nuclei counted for each colony.

### 4.5. Clonogenic survival assay

iPSCs were dissociated to single cells and 2000 WT or 4000 *MCM10^+/−^* cells were plated in 6-well plates in triplicate for each biological replicate. Qualitative images were plated at 2000 WT and *MCM10^+/−^* cells per well in a 6-well plate. For the first 48 h cells were cultured in the presence of ROCKi and then cultured as usual for a total of 6 days. Next, medium was removed, colonies were gently washed with PBS, fixed with 10% acetic acid/10% methanol solution in PBS and stained with crystal violet. Excess stain was washed off with distilled water. Plates were scanned using an Epson Expression 1680 scanner and FIJI version 1.8.0_172 was used to count the number of colonies.

### 4.6. Fluorescent activated cell sorting analysis

For analyses during the differentiation protocol, cells were washed in fluorescent activated cell sorting (FACS) buffer (1% BSA in PBS) and stained with the indicated fluorochrome-conjugated antibodies: CD3-APC (BioLegend 300412, RRID:AB_314066), CD16-FITC (BioLegend 302006, RRID:AB_314206), CD56-PerCP/Cy5.5 (BioLegend 318322, RRID:AB_893389), and/or CD34-PE (BD Biosciences 555822, RRID:AB_396151) at manufacturer recommended dilutions for 30 min at 4°C. Following surface staining, cells were washed 3 times in PBS and stained to assess viability (LIVE/DEAD Fixable Aqua Dead Cell Stain Kit, ThermoFisher L34965) for 30 min at 4°C. Excess LIVE/DEAD stain was removed by washing cells 3 times with FACS buffer. Flow cytometry data were obtained on an LSR II instrument (BD Biosciences) with 20 mW Blue (488 nm), 40 mW Red (640 nm), 25 mW Violet (405 nm) lasers and analysed by FlowJo version v10.8.1_CL (BD Life Sciences) [81].

For analyses of apoptosis, two days prior to FACS, cells were dissociated and 200 000 cells were plated per well of a 6 well plate in medium containing ROCKi. After 24 h, medium was exchanged for fresh medium without ROCKi. After 24 h, medium containing dead and dying cells and adherent cells was collected and washed with PBS. For Annexin V and PI staining to determine apoptosis rates, the APC Annexin V apoptosis kit from BioLegend (640932) was utilized following manufacturer instructions. Briefly, cells were resuspended in Annexin V binding buffer and stained simultaneously with APC Annexin V and PI solution for 15 min at room temperature. Flow cytometry data were obtained on an LSR II instrument (BD Biosciences) with 20 mW Blue (488 nm), 40 mW Red (640 nm), 25 mW Violet (405 nm) lasers and analysed by FlowJo version v10.8.1_CL (BD Life Sciences) [81].

For flow cytometry analyses of cell cycle distribution, DNA synthesis and origin licensing, nuclei were isolated and stained for flow cytometry as previously described in Matson *et al*. [82]. Briefly, cells were pulsed with 10 µm Edu for 30 min before harvesting. Following harvesting, cells were extracted on ice with CSK buffer (PIPES 10 mM, sucrose 300 mM, NaCl 100 mM and MgCl_2_ 3 mM) containing 0.5% Triton X-100, and protease/phosphatase inhibitors (1 mg ml^−1^ pepstatin A, 1 mg ml^−1^ leupeptin, 1 mg ml^−1^ aprotinin, 10 mg ml^−1^ phosvitin, 1 mM β-glycerol phosphate, and 1 mM sodium orthovanadate). Nuclei were pelleted and then fixed in 4% paraformaldehyde. Following fixation, EdU was subjected to click reaction with Alexa Fluor 647 Azide antibody (Invitrogen A10277). Primary antibody staining for MCM2 (mouse anti-MCM2, BD Biosciences 610700, RRID:AB_2141952; 1:200) was completed for 1 h at 37°C followed by secondary staining (donkey anti-mouse AF488 Jackson ImmunoResearch Labs 715-545-150, RRID:AB_2340846; 1:10 000) for 1 h at 37°C. After washing, cells were resuspended in 1% BSA, 0.1% NP-40, 1 µg ml^−1^ DAPI and 100 µg ml^−1^ RNase in PBS. The next day flow cytometry data were acquired on a BD LSR II with 20 mW Blue (488 nm), 17 mW Red (633 nm), 25 mW Violet (405 nm) lasers and data analysed by FlowJo version v10.8.1_CL (BD Life Sciences) [81].

### 4.7. Telomere restriction fragment analysis

Cells were rinsed in 100 mM Tris and 100 mM EDTA, pH 8.0, pelleted and stored at −80°C. The Gentra Puregene Cell Kit (Qiagen 158445) extraction protocol was used to isolate genomic DNA with the use of 20% SDS for cell lysis instead of the provided reagent. To confirm that DNA was not degraded, approximately 0.25 µg was fractionated on a 1% TAE gel. To assess telomere length, genomic DNA was digested with HinfI (NEB R0155) and RsaI (NEB R0167) and intact telomeric DNA purified by phenol/chloroform extraction. Telomeric DNA was resolved on a 0.7% 1× TBE agarose gel overnight at 35 V. The gel was washed in 0.25 M HCl for 20 min to depurinate, followed by denaturation with 1× denaturation solution (1.5 M NaCl, 0.5 M NaOH) and neutralization in 1× neutralization buffer (1 M Tris, 1.5 M NaCl, pH 7.5). The DNA was transferred overnight via capillary action to a Hybond-XL membrane (GE Healthcare RPN303S). The membrane was prehybridized in Church buffer for 1 h at 55°C to prevent nonspecific binding. To detect telomeric DNA, a C-rich telomere probe ((C_3_TA_2_)_4_) was labelled with ^32^P-g-ATP and used for hybridization overnight at 55°C in Church buffer. After 3 washes in 4× SSC and 1 with 4× SSC with 0.1% SDS, the membrane was exposed to a phosphor imaging screen and detection was completed with a Typhoon FLA 9500 imager. Determination of the peak of signal was completed in FIJI version 1.8.0_172 and Photoshop21.1.1 [80].

### 4.8. Induced pluripotent stem cell differentiation

For differentiation of iPSCs to NK cells we utilized the STEMdiff NK Cell Kit from Stemcell Technologies (100-0710) following the manufacturer's protocol. Briefly, to form embryoid bodies (EBs), 3.5 × 10^6^ or 0.7 × 10^6^ dissociated iPSCs were plated in a 6- or 24-well AggreWell 400 plate (34421 or 34411) respectively. Cells were cultured in EB formation medium (EB Medium A with ROCKi) for the first 2 days followed by half medium change with EB Medium A on day 2 and EB Medium B on day 3. On day 5, EBs were harvested utilizing a 37 µm filter (Stemcell Technologies 27215) and transferred to a non-tissue culture treated plate (Stemcell Technologies 100-0096 or 100-0097) with EB Medium B. Half medium changes were completed every 2 to 3 days with EB Medium B. On day 12, EBs were harvested and dissociated by incubating with collagenase II (Stemcell Technologies 07418) for 20 min followed by TrypLE Express (Gibco 12604-021) for 20 min. Any additional dissociation was completed mechanically by pipetting up and down. Collagenase and TrypLE were removed, and cells were resuspended in PBS containing 2% FBS and 1 mM EDTA. To assess total number of hematopoietic stem cells generated by day 12, total cell number of dissociated cells was determined with Trypan Blue (Invitrogen T10282) on Countess slides (Invitrogen C10283) using a Countess automated cell counter (Invitrogen C20181). A sample of this cell suspension was stained for CD34. After this sample was set aside, CD34^+^ cells were isolated using positive immunomagnetic selection (EasySep Human CD34-Positive Selection Kit II, Stemcell Technologies 17856) as directed by the manufacturer with 2 rounds of magnetic isolation. The purity of CD34^+^ cells was determined by flow cytometry and the total number of CD34^+^ cells in the purified sample was determined based on cell counts with a Countess automated cell counter and Trypan Blue. To generate LPs, 25 000 CD34^+^ cells were plated per well of a 24-well plate. This 24-well plate was non-tissue-culture treated and previously coated with lymphoid differentiation coating material (component of STEMdiff NK Cell Kit from Stemcell Technologies). The cells were cultured in LP medium with half medium changes every 3 to 4 days. Cells were transferred to a freshly coated plate on day 7. On day 14, cells were collected and counted with a Countess automated cell counter and Trypan Blue. 50 000 cells were plated per well of a tissue-culture treated 24-well plate irrespective of surface markers. Cells were cultured for an additional 14 days in the NK Cell Differentiation medium. Half medium changes were performed 3 to 4 days and if needed cells were split in half.

### 4.9. Natural killer cell functional assays

NK effector cells were plated alone or with target K562 cells at a 2:1 ratio. The cells were incubated with anti-CD107a (BioLegend 328606, RRID:AB_1186036) at 37°C and 5% CO_2_. After 1 h, cytokine release was inhibited with Golgi Stop (BD Biosciences 554724, RRID:AB_2869012) and Golgi Plug (BD Biosciences 555029, RRID:AB_2869014). The cells were incubated together for an additional 4 h. At collection, surface staining for CD3 (BioLegend 317330, RRID:AB_2563507), CD56 (BioLegend 92189), and CD45 (BioLegend 304042, RRID:AB_2562106) was performed followed by fixation with 2% paraformaldehyde and permeabilization with 0.1% Triton X. Intracellular staining was then completed for INF*γ* (BioLegend 93705) and TNF*α* (BioLegend 92960). Samples were analysed with a LSR II instrument (BD Biosciences) with 20 mW Blue (488 nm), 40 mW Red (640 nm), 25 mW Violet (405 nm) lasers and data analysed by FlowJo version v10.8.1_CL (BD Life Sciences) [81].

### 4.10. Fluorescence *in situ* hybridization

For T-FISH of metaphase spreads, cells were arrested in metaphase with 0.1 µg ml^−1^ colcemid (KaryoMAX, ThermoFisher 15212012) for 3 h. After arrest, cells were resuspended in 0.075 M KCl for 30 min followed by drop-wise addition of fixative (3:1 methanol:acetic acid) and incubation of 10 min. Cells were then pelleted and underwent an additional 3 rounds of fixation. After fixation cells were dropped onto slides and allowed to dry. Prior to staining, slides were rehydrated in PBS and fixed with 3.7% formaldehyde followed by dehydration via ethanol series. T-FISH was performed with TelC-Cy3 probe (PNA bio F1002) and denaturation of DNA by heating to 80°C. Hybridization was allowed to complete overnight at 4°C. Slides were washed twice with PNA A (70% formamide, 0.1% BSA, 10 mM Tris, pH 7.2) and 3 times in PNA B (100 mM Tris, pH 7.2, 150 mM NaCl, 0.1% Tween-20). The second PNA B wash contained DAPI. Slides were dehydrated, dried and mounted with Vectashield (Vector Laboratories H-1000). Blinded slides were imaged using a Zeiss spinning disc confocal microscope. To quantitatively measure telomere fluorescence intensity, FIJI version 1.8.0_172 was used [80]. Z-stack images were split into respective channels and flattened using a Sum Slices projection. An 8-bit duplicate of the telomere image was used to generate a mask for telomere identification. The automatic threshold moments methodology was applied to eliminate background and the image was converted to a binary image [83]. Telomere occupied regions were automatically detected as regions of interest (ROIs). These ROIs were manually confirmed to contain a single telomere or were removed from the ROI list. This ROI mask was applied to the original flattened image and integrated density was measured at each ROI as a measure of intensity. To assess signal free ends, the ROI mask was overlayed on the original image and chromosome ends examined to determine if a telomere was not detected by the automatic threshold.

For T-FISH of interphase cells, two days prior to staining, cells were dissociated, and 50 000 cells were plated per well of a chamber slide (Falcon 354114) in medium containing ROCKi. After 24 h, medium was exchanged for medium without ROCKi. After an additional 24 h, cells were fixed with 4% paraformaldehyde (Electron Microscopy Sciences 15714) for 10 min at room temperature and permeabilized with 0.1% Triton X-100 for 5 min at room temperature. Cells were blocked in ABDIL (20 mM Tris, pH 7.5, 2% BSA, 0.2% fish gelatin, 150 mM NaCl, 0.1% sodium azide) with 100 µg ml^−1^ RNase A for 1 h at room temperature. Slides were then dehydrated via ethanol series and T-FISH performed as described above. For centromere FISH, samples were co-stained with TelC-Cy3 (PNA bio F1002) and CENPB-Alexa488 (PNA bio F3004) and processed as described above. Example images were acquired on a Zeiss spinning disc confocal microscope.

### 4.11. Statistical analysis

PRISM software was utilized for statistical analysis of the data. Test type and *p* values are indicated in the figure legend of each experiment.

## Data Availability

Supplementary material is available online [84].
